# NKX6.3 Regulates Reactive Oxygen Species Production by Suppressing NF-kB and DNMT1 Activities in Gastric Epithelial Cells

**DOI:** 10.1038/s41598-017-02901-y

**Published:** 2017-06-05

**Authors:** Jung Hwan Yoon, Olga Kim, Suk Woo Nam, Jung Young Lee, Won Sang Park

**Affiliations:** 1 Department of Pathology, College of Medicine, The Catholic University of Korea, 505 Banpo-dong, Seocho-gu Seoul, 137-701 South Korea; 2 Department of Functional RNomics, College of Medicine, The Catholic University of Korea, 505 Banpo-dong, Seocho-gu Seoul, 137-701 South Korea

## Abstract

NKX6.3 plays an important role in gastric epithelial differentiation and also acts as a gastric tumor suppressor. The specific aim of this study was to determine whether NKX6.3 contributes to gastric mucosal barrier function by regulating reactive oxygen species (ROS) production. NKX6.3 reduced ROS production and regulated expression of anti-oxidant genes, including *Hace1*. In addition, NKX6.3 reduced DNMT1 expression and activity by down-regulating *NF-kB* family gene transcription. Silencing of Hace1 recovered ROS production, whereas knock-down of DNMT1 and NF-kB reduced ROS production and induced Hace1 expression by hypomethylating its promoter region. In addition, NKX6.3 inhibited CagA effects on cell growth, ROS production, and NF-kB and DNMT1 activity. In gastric mucosae and cancers, *NKX6*.*3* and *Hace1* expression was significantly reduced. The *NKX6*.*3* expression was positively correlated with *Hace1* and *Nrf2* genes, but negatively correlated with *DNMT1*. Hypermethylation of *Hace1* gene was observed only in gastric mucosae with *H*. *pylori*, atrophy and intestinal metaplasia. Thus, these results suggest that NKX6.3 inhibits ROS production by inducing the expression of Hace1 via down-regulating NF-kB and DNMT1 activity in gastric epithelial cells.

## Introduction

Gastric cancer has a high incidence in Asia and is a leading cause of cancer death in the region^1^. Cellular damage from various agents and chronic infection with *H*. *pylori* most likely influence the risk of gastric carcinogenesis through inflammation-induced reactive oxygen species (ROS) and reactive nitrogen species (RNS) leading to DNA damage in gastric epithelial cells and precancerous cascade including atrophic gastritis, intestinal metaplasia and dysplasia^2–4^.

The ROS, including superoxide anion radical (•O_2_^−^), hydrogen peroxide (H_2_O_2_), and hydroxyl radical (•OH), are highly reactive, diffusible, and ubiquitous molecules generated as inevitable by-products of aerobic respiration and metabolism^5^. In stomach, *H*. *pylori* infection induces a strong inflammatory host response, leading to the generation of several ROS and RNS via neutrophils and macrophages^6^. Imbalance between ROS production and the capacity for detoxification induces an alteration of gene expression^7^, increased cell death and proliferation, and induction of DNA mutation^8^. Notably, cancer cells augment intracellular ROS, which contributes to tumorigenesis and cancer progression by promoting genomic instability through increased DNA damage and reduced mismatch repair^9^. However, as excessive levels of ROS stress can induce cancer cell cycle arrest and apoptosis, cancer cells also increase expression of anti-oxidant proteins to detoxify from ROS and anti-apoptotic proteins^10^. Thus, manipulating the unique redox regulatory mechanisms of cancer cells might be an effective strategy to eliminate these cells^11^.

Previously, we and others have reported that the novel transcription factor, NKX6.3^12^, is a key factor for gastric differentiation and prevention of intestinal metaplasia and acts as a tumor suppressor for gastric cancer^13–16^. ROS is reportedly involved in the development of precancerous gastritis and gastric cancer, hence, we hypothesized that NKX6.3 may protect the gastric mucosal epithelia from harmful ROS.

Herein, we examined the effects of NKX6.3 on ROS production and Hace1, Nrf2, mnSOD, catalase, GSH, Nqo1, and Ho-1 expression in mock and NKX6.3 stable AGS and MKN1 cells. In addition, the expression of NKX6.3, Hace1, Nrf2, and DNMT1 was compared between non-neoplastic gastric mucosae and gastric cancers. Overall, we found that NKX6.3 significantly decreased ROS production and regulated the expression of ROS-related genes, including *Hace1*, by suppressing DNMT1 and NF-kB activity. These results suggest that NKX6.3 plays an important role in gastric epithelial protection and gastric cancer prevention by regulating the ROS levels.

## Results

### NKX6.3 attenuates ROS production by regulating ROS-responsive genes

**Figure 1:**
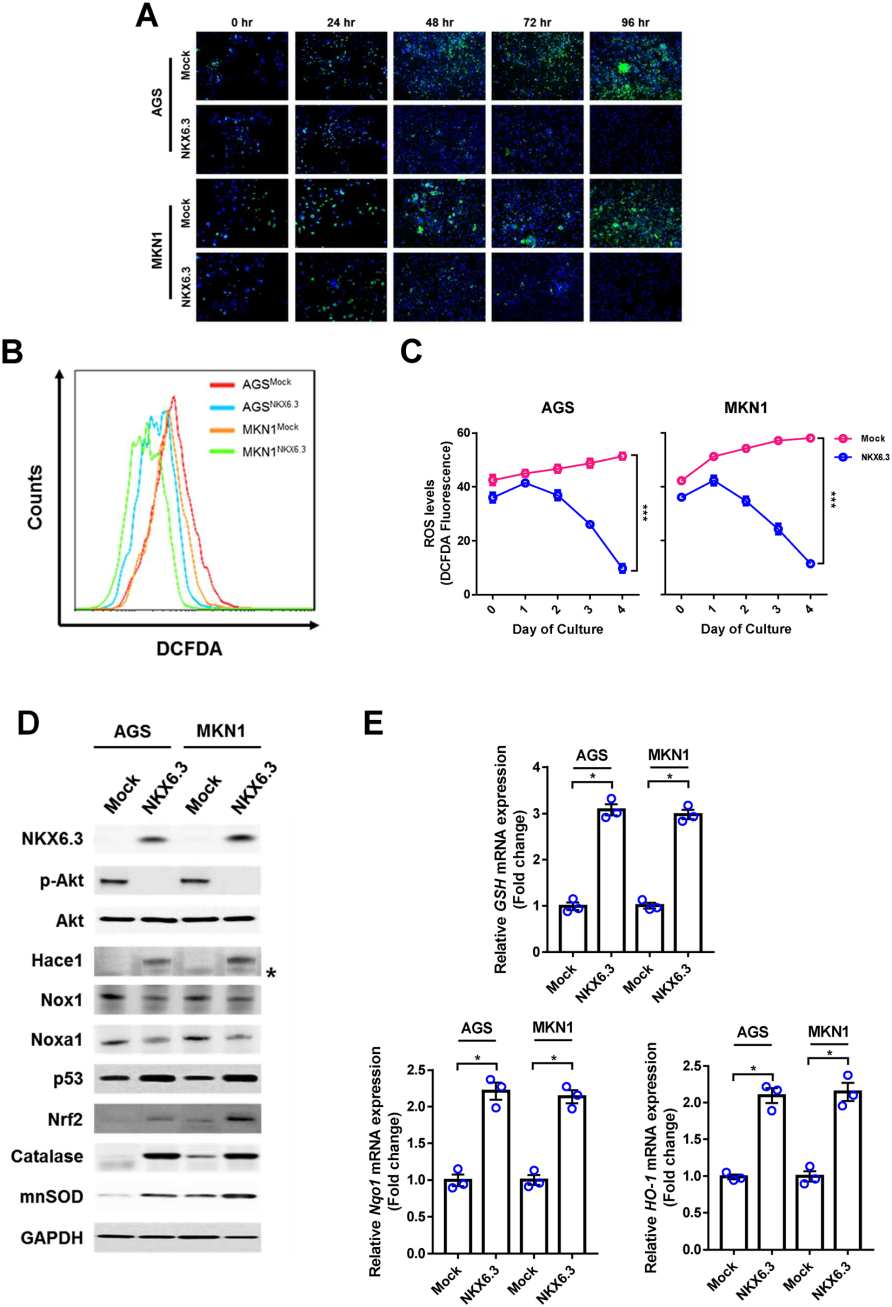
NKX6.3 reduces ROS levels by regulating ROS-responsive genes. **(A–C)** Measurement of ROS using Fluorescence microscopy and FACS analysis by DCFDA staining. Stable expression of NKX6.3 in AGS and MKN1 cells showed reduced levels of ROS in a time-dependent manner, compared to mock stable AGS and MKN1 cells. Results are represented as mean ± SEM from three independent experiments. **(D)** NKX6.3 significantly induced the expression of Hace1, Nrf2, catalase and mnSOD, and reduced the expression of Nox1 and Noxa1 proteins in western blot assay. **(E)** In real-time PCR analysis, NKX6.3 induced *GSH*, *Nqo-1* and *Ho-1* mRNA transcript. Results are represented as mean ± SEM from three independent experiments. Each dot plot represents the result from the individual experiment. ***P < 0.0001, *P < 0.05, based on the student’s t-test.

**Figure 2:**
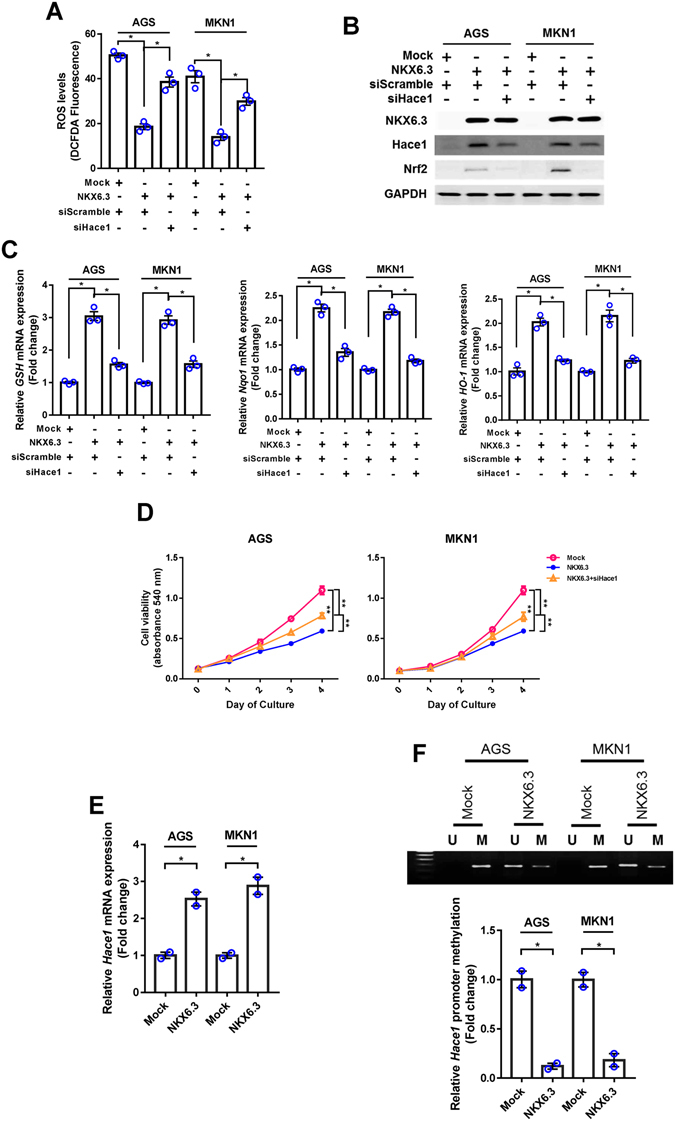
NKX6.3 increases Hace1 expression by inducing demethylation of *Hace1* promoter. **(A)** In DCFDA staining analysis, knock-down of Hace1 partially recovered ROS production in NKX6.3 stable cells. Results are represented as mean ± SEM from three independent experiments. **(B)** In NKX6.3 stable cells, knock-down of Hace1 reduced Nrf2 expression in western blot analysis. **(C)** In real-time PCR analysis, Hace1 silencing resulted in decreased *GSH*, *Nqo1* and *Ho-1* mRNA expression in NKX6.3 stable cells. Results are represented as mean ± SEM from three independent experiments. **(D)** NKX6.3 stable cells showed time-dependent inhibition of cell viability, but knock-down of Hace1 in NKX6.3 stable cells showed recovery of cell viability. Results are represented as mean ± SEM from three independent experiments. **(E)** NKX6.3 induced the expression of *Hace1* mRNA transcript in AGS and MKN1 cells in real-time PCR. Results are represented as mean ± SEM from three independent experiments. **(F)** Hypermethylation of CpG island at *Hace1* promoter was found in mock stable AGS and MKN1 cells, whereas NKX6.3 expression induced demethylation of *Hace1* promoter in MSP and real-time qPCR. Results are represented as mean ± SEM from three independent experiments. Each dot plot represents the result from the individual experiment. **P < 0.01, *P < 0.05, based on the student’s t-test.

To determine whether NKX6.3 contributes to ROS production, we performed *in vitro* ROS analysis in AGS^Mock^, MKN1^Mock^, AGS^NKX6.3^ and MKN1^NKX6.3^ cells using DCFDA staining assay. Stable NKX6.3 expression in AGS and MKN1 cells showed reduced cellular ROS levels in a time-dependent manner, as compared to mock stable AGS and MKN1 cells (Fig. 1A–C). To further confirm these initial observations, we measured the expression levels of ROS-related genes, including Hace1, Nox1, Noxa1 and Nrf2, and oxidative stress-responsive genes, including catalase, mnSOD, GSH, Nqo1 and Ho-1 by real-time RT-PCR and western blotting. Interestingly, NKX6.3 induced the expression of Hace1, Nrf2, catalase, and mnSOD, while decreasing the expression of Nox1 and Noxa1 proteins. In addition, it also reduced the expression of *GSH*, *Nqo1* and *Ho-1* at the mRNA level (Fig. 1D and﻿ E). To further support our results, we recapitulated *NKX6*.*3*, *Hace1* and *Nrf2* gene expression patterns from large cohorts of gastric cancer patients available from the National Center for Biotechnology Information (NCBI) Gene Expression Omnibus (GEO) database (accession numbers GSE27342). Interestingly, *NKX6*.*3* expression was positively correlated with *Hace1* and *Nrf2* expression in gastric cancer cohort (Supplementary Figure S1A). In addition, *Hace1* and *Nrf2* expression showed a positive correlation (Supplementary Figure S1A). Next, we aimed to determine whether the anti-oxidant activity of NKX6.3 is dependent on Hace1 expression. In AGS and MKN1 cells, treatment with *siHace1* partially recovered ROS production (Fig. 2A) and markedly reduced Nrf2 expression (Fig. 2B). Also, Hace1 silencing in NKX6.3 stable cells decreased *GSH*, *Nqo1* and *Ho-1* mRNA expression (Fig. 2C) and showed moderate ablation of NKX6.3-induced cell growth inhibition (Fig. 2D). Thus, it is likely that NKX6.3 attenuates ROS production by Hace1-dependent or –independent regulation of ROS-mediated gene expression.

### NKX6.3 induces expression of Hace1-HECT E3 ligase

**Figure 3:**
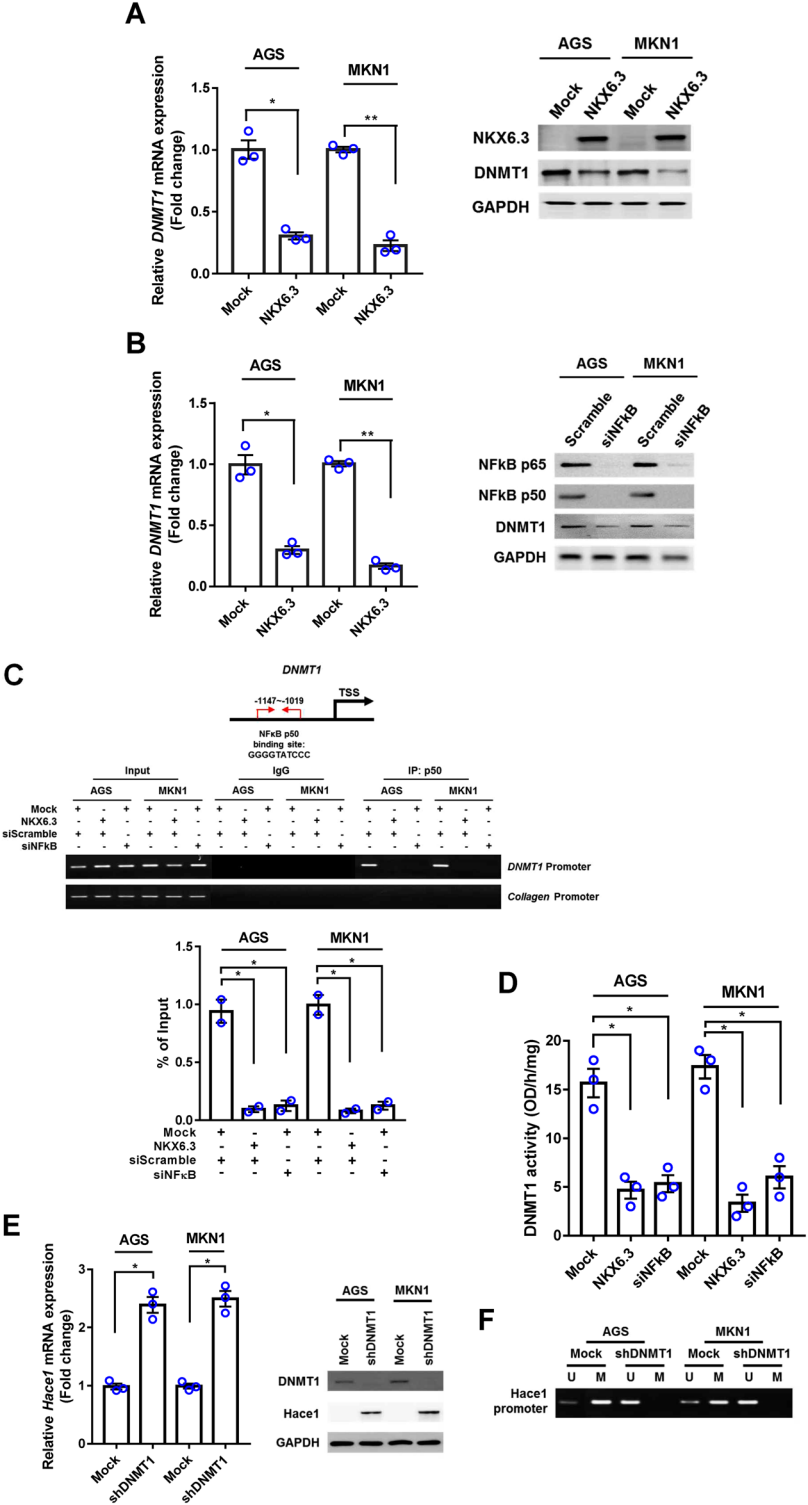
NKX6.3 inhibits the expression and activity of DNMT1. **(A)** NKX6.3 reduced DNMT1 mRNA and protein expression in AGS and MKN1 cells. **(B)** Inactivation of NF-kB significantly decreased DNMT1 mRNA and protein expression in AGS and MKN1 cells. **(C)** In ChIP assay, NKX6.3 inhibited p50 binding to the promoter region of *DNMT1* gene and its inhibitory effect was very similar to that of *siNF-kB*. Collagen gene was used as a negative control and bands were seen in the input but not in the IgG and p50 precipitated genomic DNA. **(D)** NKX6.3 expression and NF-kB silencing significantly reduced DNMT1 activity. **(E)** Inactivation of DNMT1 with *shDNMT1* induced Hace1 mRNA and protein expression. **(F)** DNMT1 silencing induced de-methylation of *Hace1* promoter. All results are represented as mean ± SEM from three independent experiments. Each dot plot represents the result from the individual experiment. **P < 0.01, *P < 0.05, based on the student’s t-test.

The Hace1-HECT E3 ligase is a tumor suppressor that directly regulates ROS production, and its reduced expression due to promoter hypermethylation is frequently observed in several cancers^17, 18^. Since NKX6.3 induced re-expression of Hace1 protein (Fig. 1D), we hypothesized that NKX6.3 functions as a hypomethylating agent. Expectedly, expression of *Hace1* mRNA was markedly increased in NKX6.3 stable transfectants (Fig. 2E). Notably, hypermethylation of CpG islands on *Hace1* promoter was observed in mock stable AGS and MKN1 cells, whereas de-methylation on *Hace1* promoter was detected in NKX6.3 stable transfectants (Fig. 2F). Next, we evaluated the regulatory role of NKX6.3 in DNMT1 expression. As shown in Fig. 3A, NKX6.3 significantly down-regulated expression of DNMT1 mRNA and protein in AGS^NKX6.3^ and MKN1^NKX6.3^ cells. Knockdown of NF-kB, a transcription factor for DNMT1^19^, with *siNF-kB* markedly decreased DNMT1 expression at the mRNA and protein levels (Fig. 3B). ChIP assay results indicated that p50 binding to the *DNMT1* gene promoter was significantly inhibited by NKX6.3, comparable with the effect of *siNF-kB* (Fig. 3C). In addition, NKX6.3 expression as well as NF-kB silencing significantly decreased DNMT1 activity (Fig. 3D). Knock-down of DNMT1 with *shDNMT1* increased Hace1 mRNA and protein expression (Fig. 3E) and induced de-methylation of *Hace1* gene in AGS and MKN1 cells (Fig. 3F). These findings collectively suggest that NKX6.3 induces Hace1 expression by suppressing DNMT1 activity.

### NKX6.3 down-regulates NF-kB expression and activity

**Figure 4:**
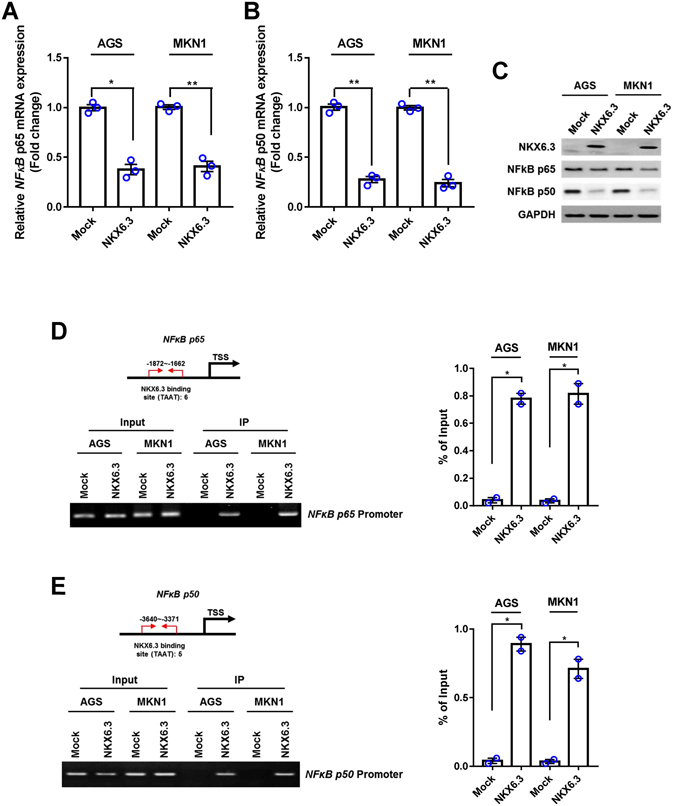
NKX6.3 negatively regulates NF-kB expression and activity. **(A)** NKX6.3 decreased the expression of NF-kB subunit *p65* mRNA transcript in real-time PCR analysis. Results are represented as mean ± SEM from three independent experiments. **(B)** NKX6.3 reduced NF-kB subunit *p50* mRNA expression in AGS and MKN1 cells. Results are represented as mean ± SEM from three independent experiments. **(C)** In AGS and MKN1 cells, NKX6.3 reduced expression of NF-kB p65 and p50 proteins in western blot analysis. (**D** and **E**) ChIP and ChIP-qPCR analyses of NKX6.3 binding to the promoters of NF-kB *p65* (D) and *p50* (E) genes. The results are represented as mean ± SEM from two independent experiments. Each dot plot represents the result from the individual experiment. **P < 0.01, * *P* < 0.05, based on the student’s t-test.

Next, we investigated whether NKX6.3 regulates NF-kB activity. Interestingly, NKX6.3 markedly down-regulated NF-kB p65 and p50 at the mRNA and protein levels (Fig. 4A–C). We performed ChIP assay, followed by PCR and real-time QPCR, to identify NKX6.3 binding activity within promoter sequences of the *NF-kB p65* and *p50* genes in AGS^Mock^, MKN1^Mock^, AGS^NKX6.3^ and MKN1^NKX6.3^ cells. We defined regions upstream of the *NF-kB p65* (between −1872 to −1662 bp) and *p50* (between −3640 to −3371 bp) genes, overlapping with the transcription start site (TSS) designated 0 bp with 6 and 5 NKX6.3 candidate binding sites (TAAT), respectively. NKX6.3 showed binding activity in these promoter regions of *p65* and *p50* in AGS and MKN1 cells (Fig. 4D and E). To further confirm that NKX6.3 down-regulates NF-kB expression, we analyzed NF-kB downstream target genes, including *interleukin (IL)-6*, *IL-1β*, and *TNF-α*. As expected, stable NKX6.3 expression markedly reduced *IL-6*, *IL-8* and *TNF-α* mRNA expression (Supplementary Figure S2), indicating that NKX6.3 inhibits DNMT1 activity by down-regulating NF-kB activity at the transcriptional level.

### Hace1 regulates NF-kB expression and activity

**Figure 5:**
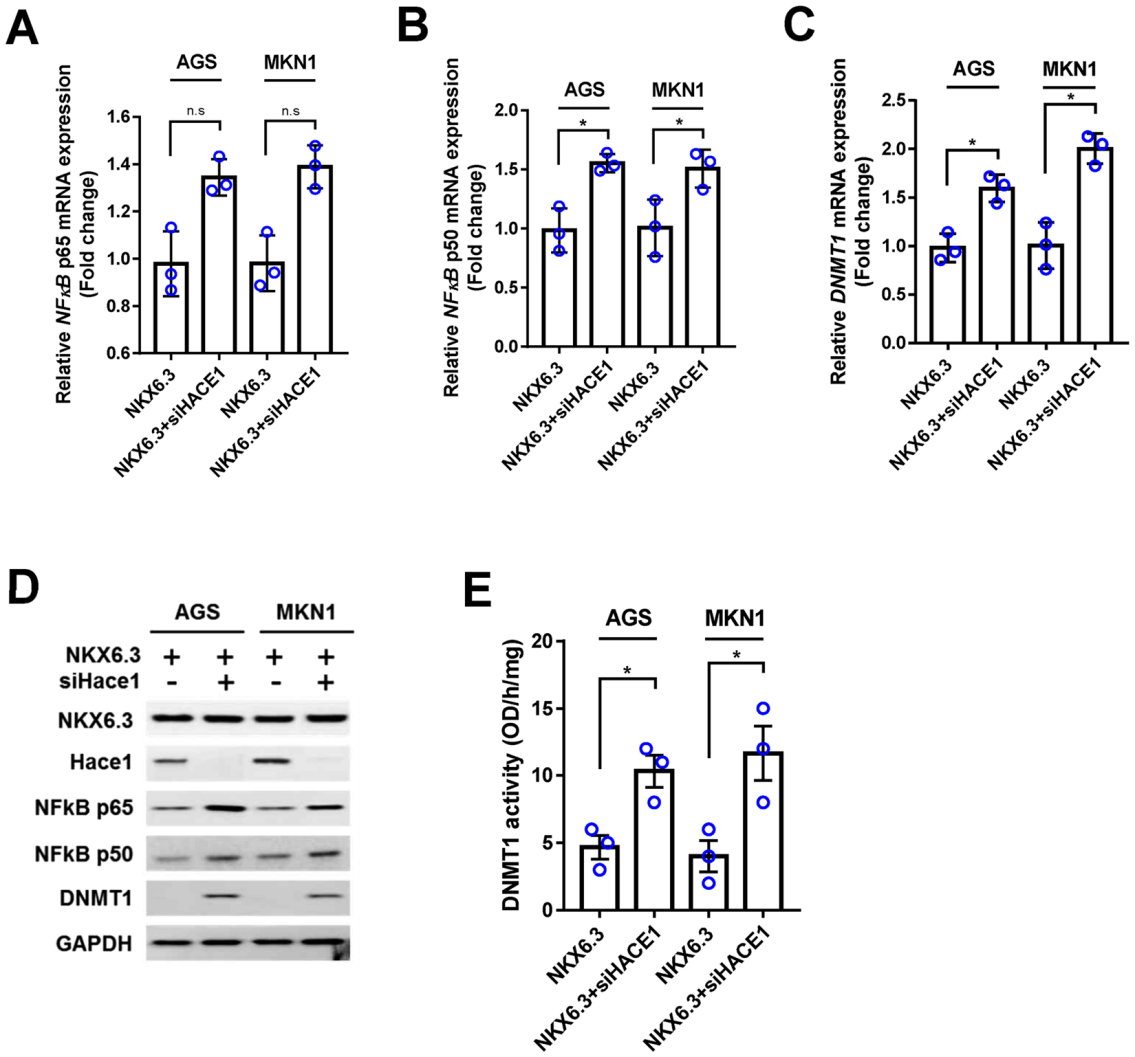
Knock-down of Hace1 induces NF-kB expression and activity. **(A–C)** Knock-down of Hace1 in NKX6.3 stable cells induced expression of NF-kB *p65* (A), *p50* (B) and *DNMT1* (C) mRNA transcript in real-time PCR analysis. Results are represented as mean ± SEM from three independent experiments. **(D)** In NKX6.3 stable cells, silencing of Hace1 with *siHace1* increased NF-kB p65, p50 and DNMT1 protein expression. **(E)** Knock-down of Hace1 induced DNMT1 activity in NKX6.3 stable cells. Results are represented as mean ± SEM from three independent experiments. Each dot plot represents the result from the individual experiment. *P < 0.05, based on the student’s t-test.

Next, we determined the regulatory role of Hace1 in NF-kB expression. As shown in Figure 5A–D, silencing of Hace1 with *siHace1* increased expression of p65, p50 and DNMT1 at the mRNA and protein levels in NKX6.3 stable transfectants. In addition, DNMT1 activity was also increased in *siHace1* transfected AGS^NKX6.3^ and MKN1^NKX6.3^ cells (Fig. 5E), suggesting that Hace1 is involved in NF-kB inactivation.

### *H*. *pylori* CagA is an important factor for ROS production

**Figure 6:**
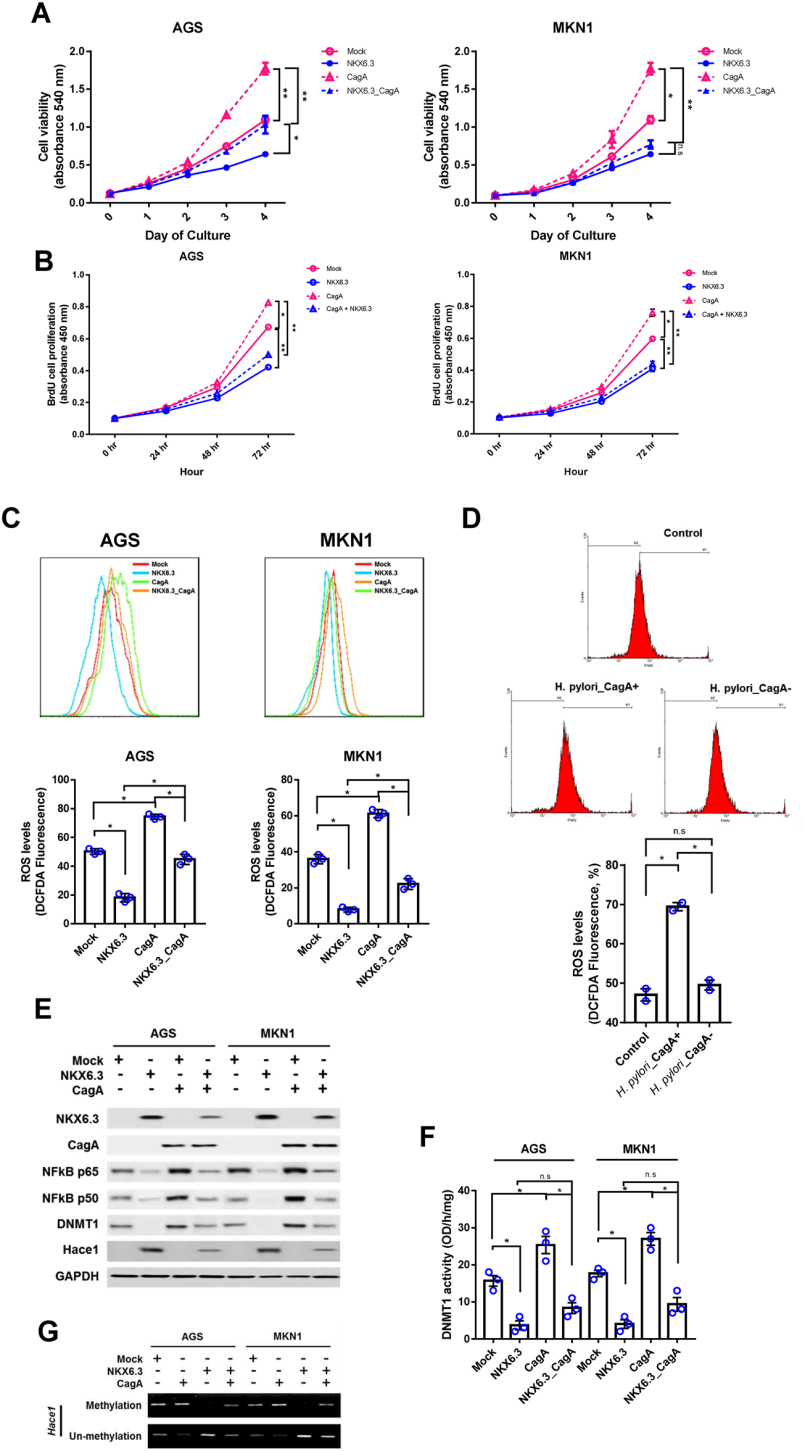
NKX6.3 suppresses the effects of *H*. *pylori* CagA on cell growth, ROS production, NF-kB signaling pathway and DNMT1 activity. (**A** and **B**) CagA induced a time-dependent increase in cell viability (A) and cell proliferation (B), but the expression of NKX6.3 inhibited the stimulatory effects of CagA on cell viability and proliferation. Results are represented as mean ± SEM from three independent experiments. **(C)** NKX6.3 abrogated CagA-induced ROS production in NKX6.3 stable cells. **(D)** CagA increased the expression of NF-kB p65, p50 and DNMT1 proteins and induced loss of Hace1 expression, but NKX6.3 markedly inhibited the CagA effects on expression of these proteins. **(E)** CagA enhanced DNMT1 activity in AGS and MKN1 cells, but NKX6.3 suppressed the CagA-induced DNMT1 activity. Results are represented as mean ± SEM from three independent experiments. **(F)** NKX6.3 inhibited CagA-induced hypermethylation of *Hace1* gene. Each dot plot represents the result from the individual experiment. **P < 0.005, *P < 0.05, based on the student’s t-test.

Here, we examined the effects of *H*. *pylori* CagA on ROS production and expression of ROS-related genes. Expectedly, CagA significantly increased cell growth and ROS production in AGS and MKN1 cells, whereas NKX6.3 ablated the effects of CagA (Fig. 6A–C). To further confirm that CagA induces ROS production, we examined ROS levels in AGS cells by using the strain of *H*. *pylori* with or without CagA. As shown in Figure 6D, *H*. *pylori* with CagA significantly increased ROS production, but *H*. *pylori* without CagA did not affect ROS levels in AGS cells. Additionally, CagA increased the expression of NF-kB p65, p50 and DNMT1 proteins and induced loss of Hace1 expression, whereas NKX6.3 markedly inhibited CagA effects on protein expression (Fig. 6E). Furthermore, NKX6.3 suppressed CagA-induced DNMT1 activity and hypermethylation of *Hace1* gene in AGS^NKX6.3^ and MKN1^NKX6.3^ cells (Fig. 6F,G). Our data suggest that NKX6.3 inactivates stimulatory effects of CagA on cell growth, ROS production, NF-kB signaling pathway, and DNMT1 activity in gastric epithelial cells.

### Expression of NKX6.3, Hace1, and DNMT1 is closely associated in gastric mucosae and cancers

**Figure 7:**
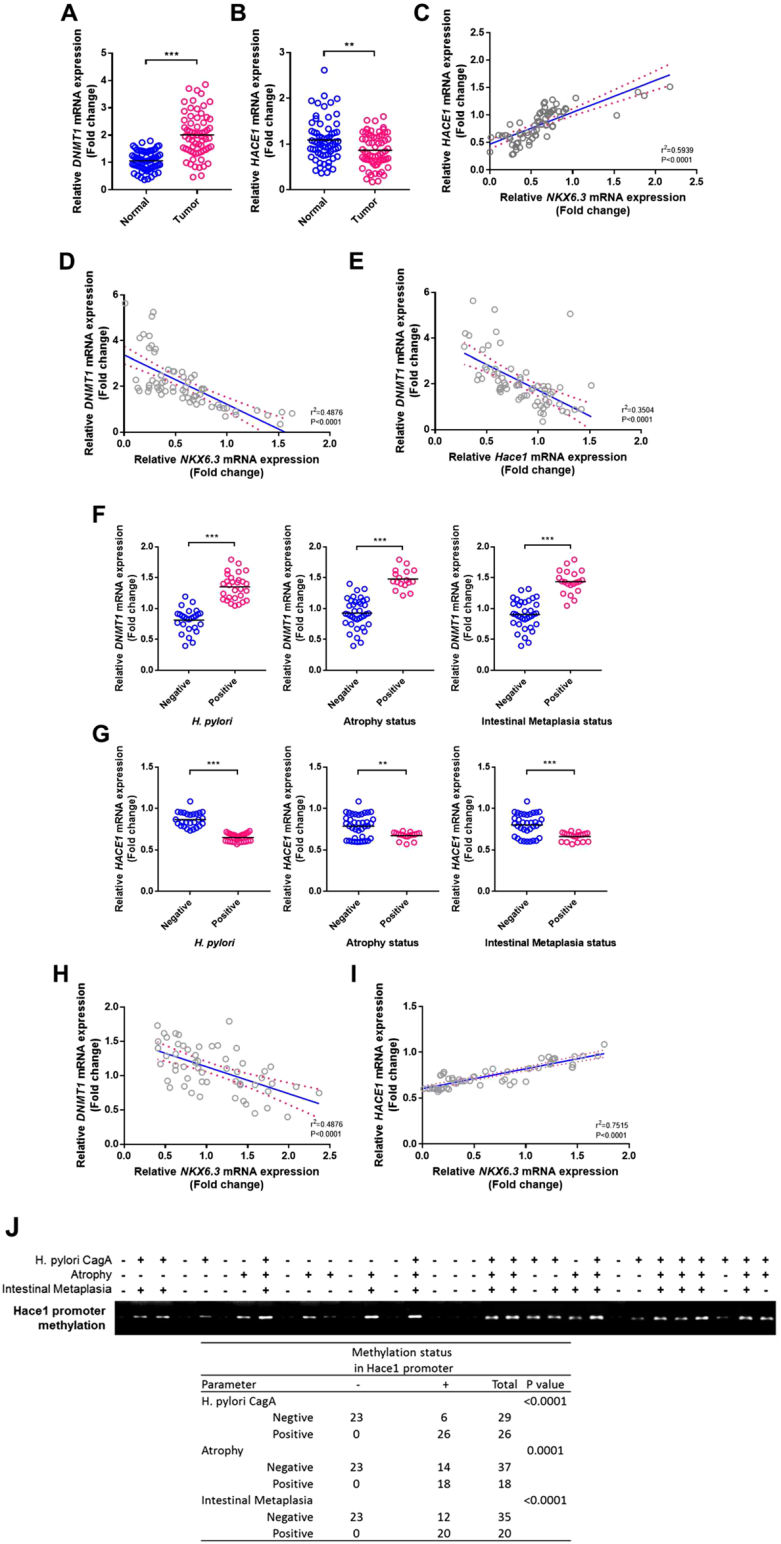
NKX6.3 is correlated with DNMT1 and Hace1 in gastric mucosae and gastric cancer tissues. (**A** and **B**) The relative mRNA expression levels of *DNMT1* (**A**) and *Hace1* (**B**) in noncancerous (Normal) and gastric cancer (Tumor) tissues are illustrated by scatterplot. The median expression level of each group is indicated by horizontal line. (**C–E**) A positive correlation of *NKX6*.*3* with *Hace1* and inverse correlation of *DNMT1* expression with *NKX6*.*3* and *Hace1* expression. (**F** and **G**) Expression levels of *DNMT1* (F) and *Hace1* (**G**) in 55 gastric mucosae with *H*. *pylori* infection, atrophy and intestinal metaplasia. (**H** and **I**) A positive correlation between *NKX6*.*3* and *Hace1* expression and inverse correlation between *DNMT1* and *NKX6*.*3* expression in non-neoplastic gastric mucosae. (**J**) Methylation status of *Hace1* promoter region examined by MSP. Hypermethylation of *Hace1* gene was observed only in the gastric mucosae with *H*. *pylori* infection, atrophy, and/or intestinal metaplasia. ***P < 0.001, **P < 0.005, based on the two-way ANOVA, linear progression and Chi-square tests.

In 65 gastric cancer tissues, *DNMT1* expression was significantly increased, whereas *Hace1* expression was significantly reduced (Fig. 7A and B). *NKX6*.*3* and *Hace1* expression were positively correlated, while *DNMT1* expression was inversely correlated with *NKX6*.*3* and *Hace1* expression in gastric cancer tissues (Fig. 7C–E). Consistently, *NKX6*.*3*, *Hace1*, and *Nrf2* expression was reduced and showed positive correlation in the large cohorts of gastric cancer patients available from the NCBI GEO database (accession numbers GSE27342; Supplementary Figure S1).

In 55 non-neoplastic gastric mucosae, *DNMT1* and *Hace1* expression was increased and reduced, respectively, in gastric mucosae with *H*. *pylori* infection, atrophy and intestinal metaplasia (Fig. 7F and G). Non-neoplastic gastric mucosae also showed positive correlation between *NKX6*.*3* and *Hace1* expression and inverse correlation between *DNMT1* and *NKX6*.*3* expression (Fig. 7H and I). The methylation status of *Hace1* promoter region was examined by MSP to confirm that reduced *Hace1* mRNA expression in gastric mucosae is caused by hypermethylation of *Hace1*. Interestingly, *Hace1* hypermethylation was observed only in the gastric mucosae with *H*. *pylori* infection, atrophy, and/or intestinal metaplasia (Fig. 7J), suggesting that NF-kB-induced DNMT1 expression caused by NKX6.3 inactivation may reduce Hace1 expression by hypermethylating the promoter region of *Hace1* in the gastric mucosa.

## Discussion

Gastric cancers develop as a consequence of chronic inflammation from persistent mucosal or epithelial cell colonization by microorganisms, including *H*. *pylori* ^20^. Chronic inflammatory cells such as macrophages/monocytes, lymphocytes, and plasma cells are present in the gastric mucosa of chronic gastritis and lead to the generation of several ROS and RNS^21^. Persistent ROS can damage cellular DNA, RNA, and proteins by chemical reactions, which subsequently cause proto-oncogene activation, oncogene/tumor suppressor gene mutations, and chromosomal aberrations^22, 23^. In stomach, *H*. *pylori* and ROS collaborate in the gastric epithelium to activate the NF-κB and AP-1 transcription factors that up-regulate the expression of chemokines, including IL-8^24, 25^, and in turn, NF-kB-dependent genes play a major role in regulating the cellular ROS levels^26^. Recently, we and others reported that NKX6.3 acts as a master regulator in gastric differentiation and proliferation^12–14^. Since the gastric mucosal barrier may protect the host from potentially harmful agents to maintain cell survival, we focused on the role of NKX6.3 in the protection of the gastric mucosal epithelia from harmful ROS.

It is well known that Hace1 functions as an important component of the cellular ROS detoxification and anti-oxidative stress responses by facilitating optimal activation of Nrf2^27^. Here, we showed that NKX6.3 reduced intracellular ROS and modulated the expression of ROS-related genes (Fig. 1). Of these, NKX6.3 induced expression of Hace1 and Nrf2 proteins in gastric cancer cells (Fig. 1D) and showed positive correlation with *Hace1* and *Nrf2* in the gastric cancer cohort (Supplementary Figure S1). In addition, Hace1 silencing with *siHace1* recovered ROS production and reduced *Nrf2*, *GSH*, *Nqo* and *Ho-1* expression (Fig. 2A–C). Furthermore, knock-down of Hace1 ablated NKX6.3-induced cell growth inhibitory activity (Fig. 2D). Taken together, these results suggest that NKX6.3 plays an important role in suppression of ROS production by regulating expression of ROS-related genes, especially *Hace1*.

NKX6.3 increased Hace1 expression and was positively correlated with *Hace1*, suggestive of direct regulation of Hace1 expression. Since Hace1 expression is significantly reduced by promoter hypermethylation in most cancer patients^17^, we investigated whether DNMT1 regulates Hace1 expression in gastric epithelial cells. NF-kB reportedly binds to one possible NF-kB binding element in the *DNMT1* promoter and DNMT1 mediates NF-kB-dependent *p16* gene promoter hypermethylation^19^. Here, we showed that NKX6.3 induced *Hace1* promoter demethylation and increased its expression (Fig. 2E and F) by down-regulating DNMT1 expression via inhibiting p50 binding in the promoter region of *DNMT1* gene (Fig. 3C). In addition, the effect of NKX6.3 on DNMT1 activity was comparable with that of *siNF-kB* treatment (Fig. 3D). Furthermore, DNMT1 silencing with *siDNMT1* led to demethylation of *Hace1* and increased its expression at the mRNA and protein levels (Fig. 3E and F). These results suggest that NKX6.3 induces Hace1 expression by inhibiting DNMT1 expression and activity.

Next, we investigated whether NKX6.3 regulates NF-kB, which is constitutively activated in most cancers^28^. NF-κB consists of a family of transcription factors, including p65 (RelA), p50 (RelB), c-Rel, p105/p50 (NF-κB1), and p100/p52 (NF-κB2), which play critical roles in inflammation, immunity, cell proliferation and survival^29^. The amino-terminus ﻿of the Rel homology domain in p50 homo- and p50/p65 heterodimers mediates specific DNA binding to the NF-κB consensus sequence present in regulatory elements of NF-κB target genes^30–32^. NKX6.3 significantly reduced p65 and p50 expression at the mRNA and protein levels (Fig. 4A–C). In particular, the promoter regions of *p65* and *p50* have 6 and 5 NKX6.3 candidate binding sites, respectively (Fig. 4D), and the binding activity of NKX6.3 to these regions was confirmed in ChIP analysis (Fig. 4E). In addition, NKX6.3 markedly reduced the expression of NF-κB target genes including *IL-6*, *IL-8* and *TNF- α* (Supplementary Figure S2). In addition, silencing of Hace1 with *siHace1* increased the expression of p65, p50 and DNMT1 and activity of DNMT1 (Fig. 5). Thus, these data indicate that NKX6.3 and Hace1 negatively control NF-kB expression and activity.

It has been reported that *H*. *pylori* induces the production of ROS and DNA damage in gastric epithelial cells and frequently causes chromosomal aberrations^33, 34^. Previously, we showed that *H*. *pylori* CagA increased the expression of NF-kB proteins and ROS levels in gastric cancer cells^35^. In the current study, CagA significantly increased cell growth and ROS production in AGS^Mock^ and MKN1^Mock^ cells, whereas NKX6.3 ablated these CagA effects by down-regulating NF-kB p65, p50 and DNMT1 and up-regulating Hace1 expression (Fig. 6A–F). These findings suggest that NKX6.3 may counteract CagA-induced NF-kB activity in gastric epithelial cells.

Damage to cellular components results in increased mutations and altered functions of important proteins in premalignant tissues, thereby contributing to the multi-stage carcinogenesis^36^. Chronic inflammation of gastric mucosa triggers a pathway of chronic gastritis, atrophy, intestinal metaplasia, dysplasia, which finally progress to gastric cancer^37^. *H*. *pylori*, especially *CagA* strains, is considered as the most important risk factor of atrophy and intestinal metaplasia^38, 39^. In gastric cancer tissue, *NKX6*.*3* and *Hace1* expression showed a positive correlation, while *DNMT1* expression was inversely correlated with *NKX6*.*3* and *Hace1* (Fig. 7A–E). The NCBI GEO database also showed reduced *NKX6*.*3*, *Hace1* and *Nrf2* expression and a positive correlation between these genes (Supplementary Figure S1). In non-neoplastic gastric mucosae with *H*. *pylori* infection, atrophy and intestinal metaplasia, *DNMT1* mRNA was increased, whereas *Hace1* mRNA was reduced. (Fig. 7F and G). *NKX6*.*3* expression was positively and inversely correlated with *Hace1* and *DNMT1*, respectively (Figure H and I). Interestingly, *Hace1* hypermethylation was observed only in the gastric mucosae with *H*. *pyori* infection, atrophy, and/or intestinal metaplasia (Fig. 7J). Thus, NKX6.3 inactivation in gastric mucosa may increase activity of NF-kB and DNMT1 and reduce Hace1 expression, subsequently progressing to atrophy, intestinal metaplasisa and cancer.

In conclusion, NKX6.3 induced Hace1 expression by suppressing DNMT1 expression and activity via down-regulating the NF-kB signaling pathway. In addition, NKX6.3 ablated CagA effects on cell proliferation, ROS production, and activities of DNMT1 and NF-kB signaling pathway. Overall, we conclude that NKX6.3 protects gastric mucosal epithelia by regulating harmful ROS production. Modulation of NKX6.3 anti-oxidant activity could contribute to the prevention of precancerous changes in gastric mucosa and gastric cancer.

## Materials and Methods

### Cell culture and generation of NKX6.3 stable cells

AGS and MKN1 gastric cancer cell lines were cultured at 37 °C in 5% CO_2_ in RPMI-1640 medium (Lonza, Basel, Switzerland) with 10% heat-inactivated fetal bovine serum (FBS). *NKX6*.*3* and *CagA* cDNAs were cloned into the pcDNA3.1 expression vectors (Invitrogen, Carlsbad, CA, USA) and *siHACE*1, *siDNMT1*, and *siNF-kB* were cloned into pSilencer neo vectors (Invitrogen, Carlsbad, CA, USA). We generated stable *NKX6*.*3* transfectants of AGS and MKN1 cells, AGS^NKX6.3^ and MKN1^NKX6.3^, stably expressing human NKX6.3, as well as mock transfectants, AGS^Mock^ and MKN1^Mock^ cells, as described previously^13^. AGS^NKX6.3^ and MKN1^NKX6.3^ cells were transfected in 60 mm-diameter dishes with the expression plasmids (2 μg total DNA) using Lipofectamine Plus transfection reagent (Invitrogen) according to the manufacturer’s recommendations. Stable expression of NKX6.3 was confirmed in AGS^NKX6.3^ and MKN1^NKX6.3^ cells by western blot analysis.

### Gastric mucosa and cancer tissue specimens

A total of 65 patients with sporadic gastric cancer who underwent a gastrectomy at the Chonnam National University Hwasun Hospital were enrolled. In addition, a total of 55 non-neoplastic frozen gastric mucosa remote (>5 cm) from gastric cancer were included in this study. Adjacent gastric mucosal tissues to each frozen specimen were also fixed in formalin and stained with hematoxylin-eosin. Informed consent was provided according to the Declaration of Helsinki. Written informed consent was obtained from all subjects. The study was approved by the Institutional Review Board of The Catholic University of Korea, College of Medicine (MC15SISI0015). There was no evidence of familial cancer in any of the patients.

Histological assessment of 55 non-neoplastic gastric mucosae was performed independently by two pathologists. According to the updated Sydney system^40, 41^, gastritis was determined by polymorphonuclear leukocyte infiltration, mononuclear cell infiltration, glandular atrophy and intestinal metaplasia, as previously described^14^. Infection with a CagA-positive strain of *H*. *pylori* was determined by the presence of CagA protein in 55 gastric mucosa tissue samples using western blot analysis, as described previously^35^.

### Measurement of cell viability and proliferation

Cell viability was determined in AGS and MKN1 gastric cancer cells after treatment with *siHace1* or *H*. *pylori CagA* transfection. To assess cell viability, a MTT [3-(4,5 dimethylthiazol-2-yl)−2,5-diphenyltetrazoliumbromide] assay was performed at 24, 48, 72, and 96 hrs following transfection with *siHace1* and *CagA* in mock, AGS^Mock^ and MKN1^Mock^ cells, and NKX6.3 stable transfectants, AGS^NKX6.3^ and MKN1^NKX6.3^ cells. Absorbance was measured at 540 nm using a spectrophotometer and viability was expressed relative to the mock control.

For cell proliferation assay, a BrdU incorporation assay was performed at 24, 48, 72, and 96 hrs following transfection with *CagA* in mock and NKX6.3 stable cells using the BrdU cell proliferation assay kit (Millipore, Billerica, MA, USA), according to the manufacturer’s instruction. Absorbance was measured using a spectrophotometer at 450 nm and proliferation was expressed relative to the mock control.

### Expression of ROS-related genes in gastric cancer cell lines and tissues

Expression of *NKX6*.*3*, *Hace1*, and *DNMT1* was examined in 65 frozen gastric cancers, corresponding non-cancerous gastric mucosal tissues and 2 gastric cancer cell lines by real-time RT-PCR and Western blot analysis. After quantification of mRNA extracted from cancer tissues and non-cancerous gastric mucosae, cDNA was synthesized using the reverse transcription kit from Roche Molecular System (Roche, Mannheim, Germany), according to the manufacturer’s protocol. For QPCR, 50 ng cDNA was amplified using Fullvelocity SYBR Green QPCR Master Mix (Stratagene, La Jolla, CA, USA) and 20 pmol/μl of each primer (forward and reverse) using Stratagene Mx 3000 P QPCR system, according techniques previously published^39^. *NKX6*.*3*, *Hace1*, and *DNMT1* mRNAs were quantified by SYBR Green Q-PCR and normalized to mRNA of the *β-actin*. Sequences of the primers are described in Supplementary Table S1. Data are reported as relative quantities according to an internal calibrator using the 2^−△△CT^ method^42^. All samples were tested in duplicate, and the mean values were used for quantification. In addition, the relation between expression levels of ROS-related genes and gastritis parameters, such as atrophy, intestinal metaplasia and *H*. *pylori* infection was also examined in 55 non-neoplastic gastric mucosae.

To investigate whether ablation of NKX6.3 is associated with ROS production, the expression of *NF-kB*, *Hace1*, *DNMT1*, *GSH*, *Nqo1*, and *Ho-1* mRNA were analyzed using real-time RT-PCR in AGS^Mock^, MKN1^Mock^, AGS^NKX6.3^, and MKN1^NKX6.3^ cells. The effects of Hace1 silencing with *siHace1* on the expression of ROS-related genes were also examined. To further confirm that NKX6.3 regulates NF-kB activity, we analyzed mRNA transcript expression of *IL-6*, *IL-8*, and *TNF-α*, which are downstream targets of the NF-kB^43^. Each mRNA was quantified by SYBR Green QPCR and normalized to mRNA of the housekeeping gene, *β-actin*. The primer sequences are described in Supplementary Table S1.

For Western blot analysis, the samples were ground to very fine powder in liquid nitrogen using a pestle and mortar, suspended in an ice-cold Nonidet P-40 lysis buffer supplemented with a 1x protease inhibitor mix (Roche). Cell lysates were separated on 10% polyacrylamide gel and blotted onto a Hybond-PVDF transfer membrane (Amersham), which had been subsequently probed with specific antibodies, and then incubated with anti-mouse IgG conjugated with horseradish peroxidase. The list of antibodies is described in Supplementary Table S2. The protein bands were detected using enhanced chemiluminescence Western blotting detection reagents (Amersham).

### Methylation status of *Hace1*

Methylation analysis was carried out in 55 frozen non-neoplastic gastric mucosae and 2 gastric cancer cell lines after transfection with NKX6.3. Methylation status of the promoter region of the *Hace1* gene was determined using sodium bisulfite treatment of the DNA followed methylation specific PCR (MSP), as described in the literature with minor modifications^17^. 5 μl of the bisulfite-modified DNA was subjected to MSP using two sets of primers for methylated and unmethylated *Hace1*. The primer sequences are described in Supplementary Table S1. PCR was performed in a total volume of 30 μl, containing 5 μl of the template DNA, 0.5 μM of each primer, 0.2 μM of each dNTP, 1.5 mM MgCI_2_, 0.4 unit of Ampli Taq gold polymerase (Perkin-Elmer) and 3 μl of 10X buffer. The reaction solution was initially denatured for 1 min at 95 °C. Amplification was carried out for 40 cycles of 30 s at 95 °C, 30 s at 58 °C and 30 s at 72 °C, followed by a final 5 min extension at 72 °C. Each PCR product was loaded directly onto 2% agarose gels, stained with ethidium bromide and visualized under UV illumination.

### Generation of *CagA* gene deleted *H*. *pylori* strains

The isogenic mutant *H*. *pylori* 26695 (*∆cagA*::*aphA)*, in which most of the *cagA gene* was replaced by a *aphA* (kanamycin resistant gene from pIP1433) cassette, was made using PCR products generated with primers “kanF” (5′-GATAAACCCAGCGAACCAT) and “aphAR” (5′-GTACTAAAACAATTCATCCAGTAA) (1402 bp; *aphA* kanamycin resistance cassette); “cagA F1” (5′-ATCGTTGATAAGAACGATAGGG)and “cagA R2” (5′-ATGGTTCGCTGGGTTTATCATTGATTGCTTCTTTGACATCGGTACCAAGCGACCCAAATAG) (552 bp, upstream of deleted *cagA* segment); “cagA F5” (5′TTACTGGATGAATTGTTTTAGTACATCAAATAGCAAGTGGTTTGGGAATGACCTACTTAACAAAATCATG-) and “cagA R6” (5′-ATTGCTATTAATGCGTGTGTGG) (425 bp; downstream of deleted *cagA* segment). Natural transformation was carried out by adding 7 μl of purified PCR product containing this Δ*cagA::aphA* allele to a lawn of cells (wild type *H*. *pylori* 26695) growing exponentially on nonselective medium, and re-streaking the population on selective medium (containing 15 μg/ml of kanamycin) after 6–8 hrs or overnight incubation to obtain transformant colonies. PCR tests and sequencing of representative kan^r^ transformants demonstrated expected replacement of *cagA* by *aphA* in each case.

### Reactive oxygen species (ROS) analysis

ROS levels were determined in mock or NKX6.3 stable AGS and MKN1 cells using 2′-7′-dichlorodihydrofluorescein diacetate (DCFDA). The AGS and MKN1 cells were incubated with 10 uM DCFDA at 37 °C for 20 min and rinsed twice with cold PBS, then trypsinized and subjected to FACScan flow cytometer. To determine the effects of *H*. *pylori* CagA on ROS production, the ROS levels were examined in *H*. *pylori* with/without CagA- infected AGS cells at 12 hrs as well as in mock and NKX6.3 stable AGS and MKN1 cells at 72 hrs after transfection with *H*. *pylori* CagA. DCF fluorescence was measured by FACS analysis and intensity was plotted against the number of cells. Additionally, cells were incubated with 10 uM DCFDA at 37 °C for 20 min and quickly washed with cold PBS, and photos of representative fluorescent fields were taken under an inverted microscope.

### Chromatin immunoprecipitation (ChIP) analysis

For assessing the NKX6.3 binding activity in the promoter region of *NF-kB p65* and *p50*, ChIP assays were performed using the Thermo Scientific Pierce Agarose ChIP kit (Thermo Scientific Pierce, Rockford, IL, USA), as previously described^13^. Briefly, AGS^Mock^, MKN1^Mock^, AGS^NKX6.3^ and MKN1^NKX6.3^ cells were cultured in a 10-cm dish for 4 days. The cells were fixed with 1% formaldehyde in PBS for 10 min, washed twice with ice-cold PBS and re-suspended in lysis buffer. Nuclei were recovered by centrifugation and MNase digestion was carried out at 37 °C for 15 min. Nuclei were lysed and the extracts were immunoprecipitated with 4 µg of antibody against NKX6.3 at 4 °C overnight. Normal rabbit IgG was used as the negative control. Protein-bound DNA was recovered using affinity chromatography purification columns according to the manufacturer’s protocol (Thermo Scientific), and 5 µl of lysed nuclei were also purified under the same procedure and used as input. DNA amplification was performed by PCR using specific primers for the promoters of *NF-kB p65*, *p50* and *DNMT1* genes, as described in Supplementary Table S1. Amplification products were separated on a 2% agarose gel.

### Measurement of DNMT1 activity

The DNMT1 activity was analyzed using the DNMT1 activity assay kit (Abcam) according to manufacturer’s instructions. Briefly, AGS and MKN1 cells were collected and suspended in PBS. After centrifugation, the pellet was lysed in lysis buffer (10 mM Tris-Hcl pH 7.5, 10 mM NaCl, 2 mM MgCl_2_) containing protease inhibitor mixture (Complete; Roche Molecular Biochemicals). Then, 6 ul of 20% NP-40 was added and the mixture was incubated for 10 min at 4 °C and centrifuged for 5 min at 3000 rpm. The supernatant was collected and the pellet containing the nuclei was resuspended in 50 μl of extraction buffer (20 mM Hepes pH 7.9, 420 mM NaCl, 1.5 mM Mgcl_2_, 0.2 mM EDTA and 10% glycerol) followed by incubation for 30 min at 4 °C and collection of the nuclear extract by centrifugation. All reactions were carried out in triplicate.

### Statistical analysis

Student’s *t*-test was used to analyze the effects of NKX6.3 on cellular ROS levels, cell viability and mRNA expression changes. Two-way ANOVA test was used to analyze the expression of *DNMT1* and *HACE1* in normal gastric mucosae and gastric cancer tissues. Linear progression test was used to analyze correlations between *DNMT1*, *HACE1* and *NKX6*.*3* expression levels in normal gastric mucosae and gastric cancer tissues. Chi-square test was used to analyze correlations between *H*. *pylori* CagA, atrophy, intestinal metaplasia and methylation status of the *HACE1* promoter. Data are expressed as means ± S.D. from at least three independent experiments. A *P*-value less than 0.05 was considered to be the limit of statistical significance. All experiments were performed in triplicate to verify the reproducibility of the findings.

## Electronic supplementary material

Supplementary information
