# Supplementary material for: NKX6.3 Regulates Reactive Oxygen Species Production by Suppressing NF-kB and DNMT1 Activities in Gastric Epithelial Cells
Source: Sci Rep. 2017 Jun 5;7:2807. doi: 10.1038/s41598-017-02901-y (PMC5459835; doi:10.1038/s41598-017-02901-y)
Supplement: Supplementary file 1 — Supplementary information [file 41598_2017_2901_MOESM1_ESM.pdf]

NKX6.3 Regulates Reactive Oxygen Species Production by Suppressing NF-kB and DNMT1 Activities in Gastric Epithelial Cells

Jung Hwan Yoon, Olga Kim, Suk Woo Nam, Jung Young Lee, Won Sang Park

Supplementary Figure S1. Expression of NKX6.3, Hace1 and Nrf2 in gastric cancer cohort

Supplementary Figure S2. NKX6.3 inhibited *IL-6, IL-8* and *TNF-α* mRNA expression

Supplementary Figure S3. Uncropped gel image for Figure 1.

Supplementary Figure S4. Uncropped gel image for Figure 2.

Supplementary Figure S5. Uncropped gel image for Figure 3.

Supplementary Figure S6. Uncropped gel image for Figure 4.

Supplementary Figure S7. Uncropped gel image for Figure 5.

Supplementary Figure S8. Uncropped gel image for Figure 6.

Supplementary Figure S9. Uncropped gel image for Figure 7.

Supplementary Table S1. List of primers used in this study

Supplementary Table S2. List of antibodies used in this study

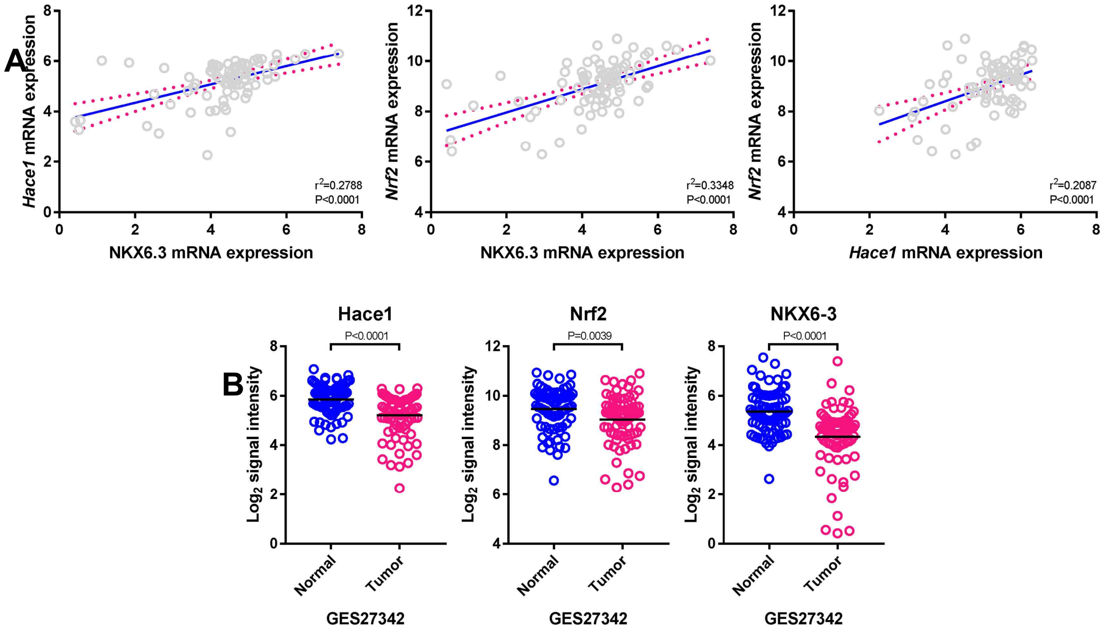

Supplementary Figure S1. Expression of NKX6.3, Hace1 and Nrf2 in gastric cancer cohort.

A. Correlation between NKX6.3, Hace1 and Nrf2 the large cohorts of gastric cancer patients. NKX6.3 expression was positively correlated with Hace1 and Nrf2 expression, and the Hace1 positively correlation with Nrf2 expression. **B.** *NKX6.3, Hace1*, and *Nrf2* expression was reduced in the large cohorts of gastric cancer patients.

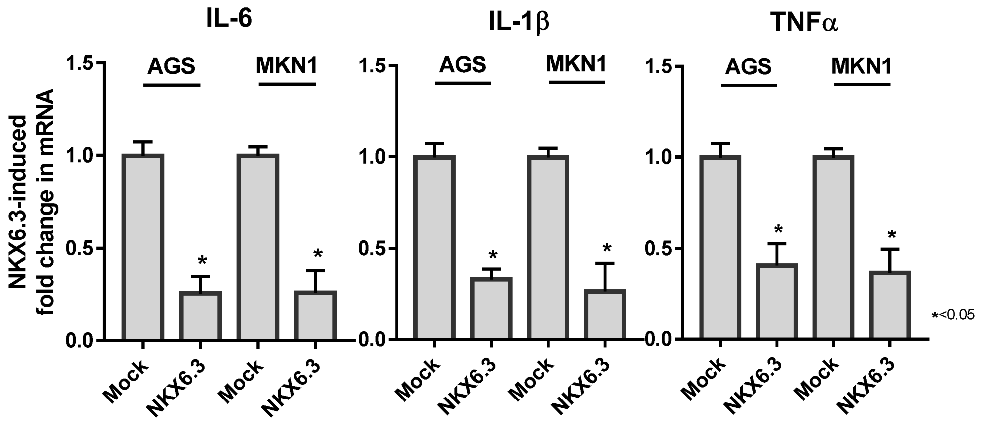

Supplementary Figure S2. NKX6.3 inhibited *IL-6, IL-8* and *TNF-α* mRNA expression.

NKX6.3 expression markedly reduced NF-kB downstream target genes, such as *IL-6, IL-8* and *TNF-α* mRNA expression.

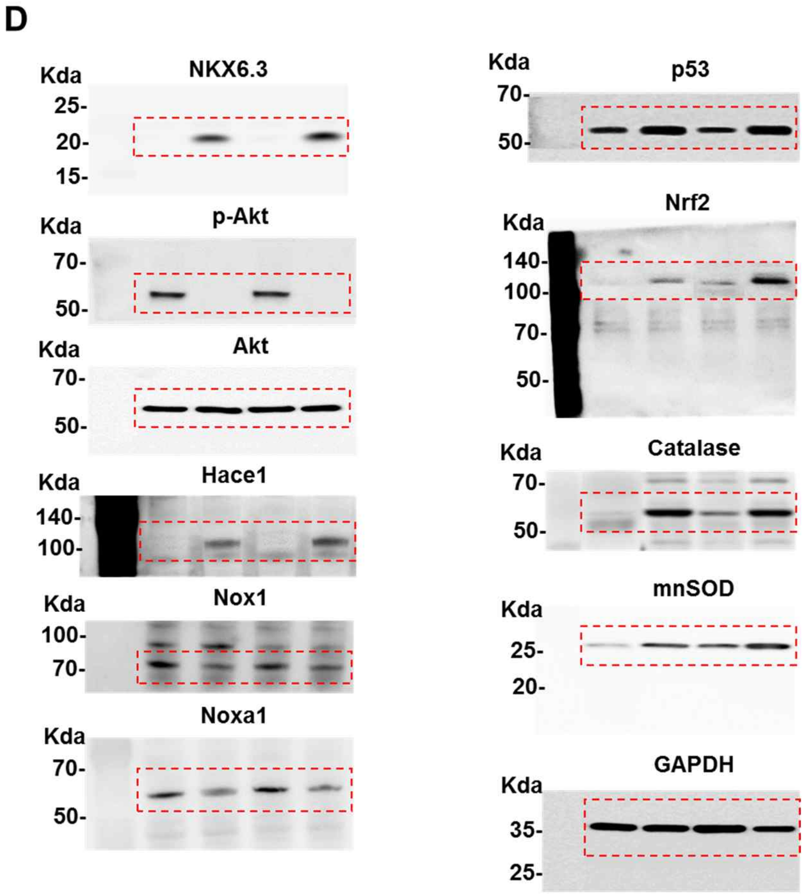

Supplementary Figure S3. Uncropped gel image for Figure 1.

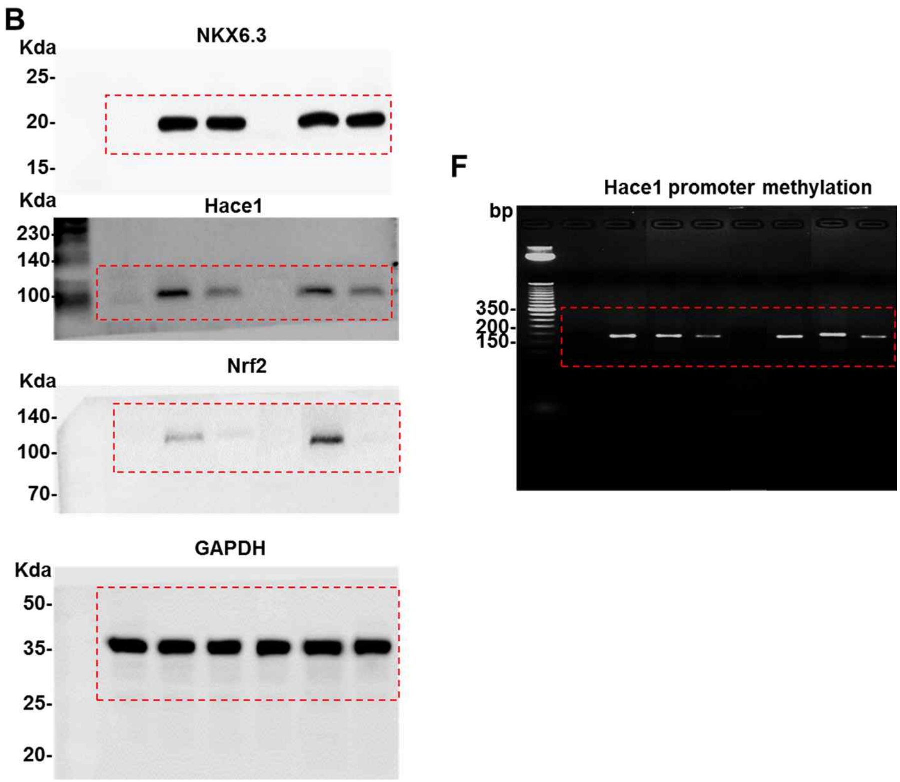

Supplementary Figure S4. Uncropped gel image for Figure 2.

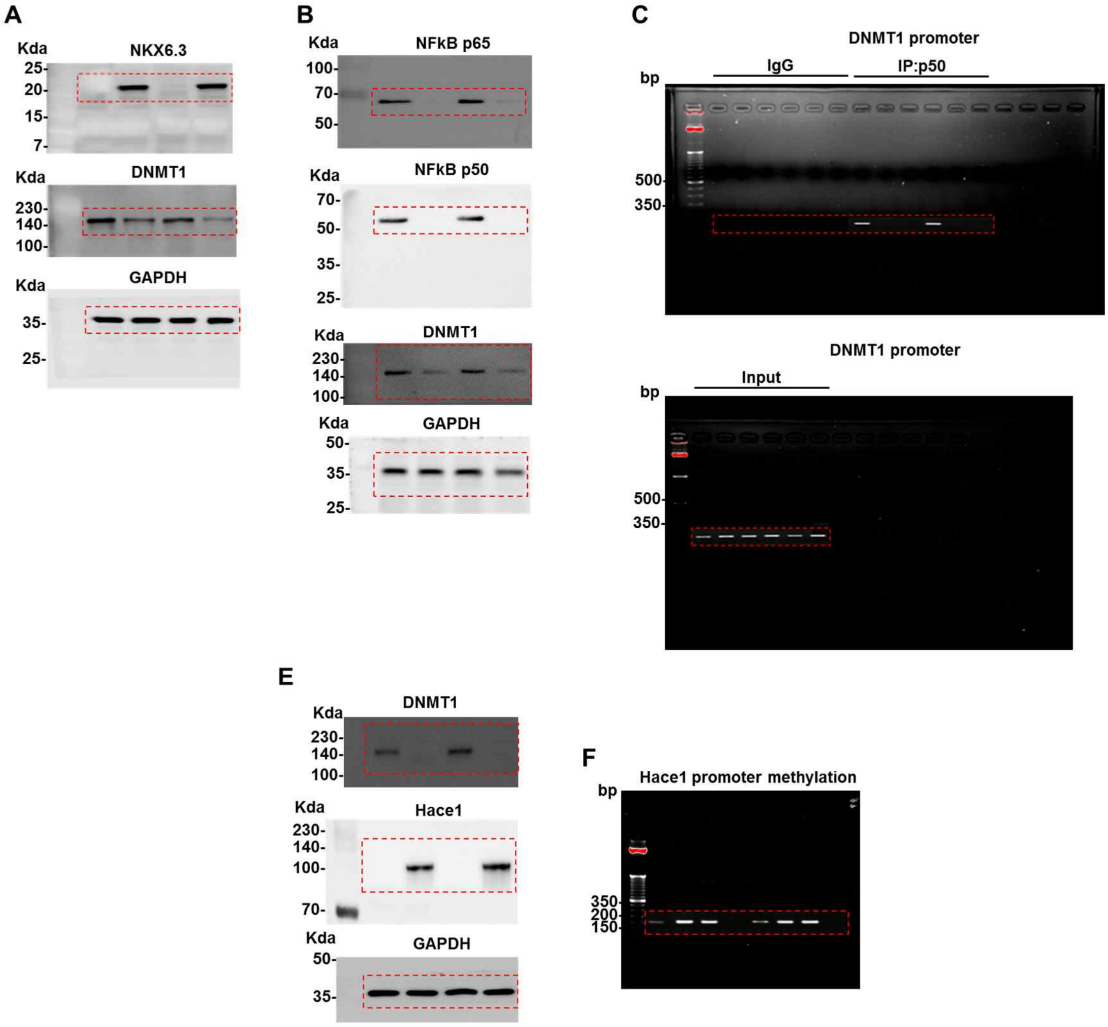

Supplementary Figure S5. Uncropped gel image for Figure 3.

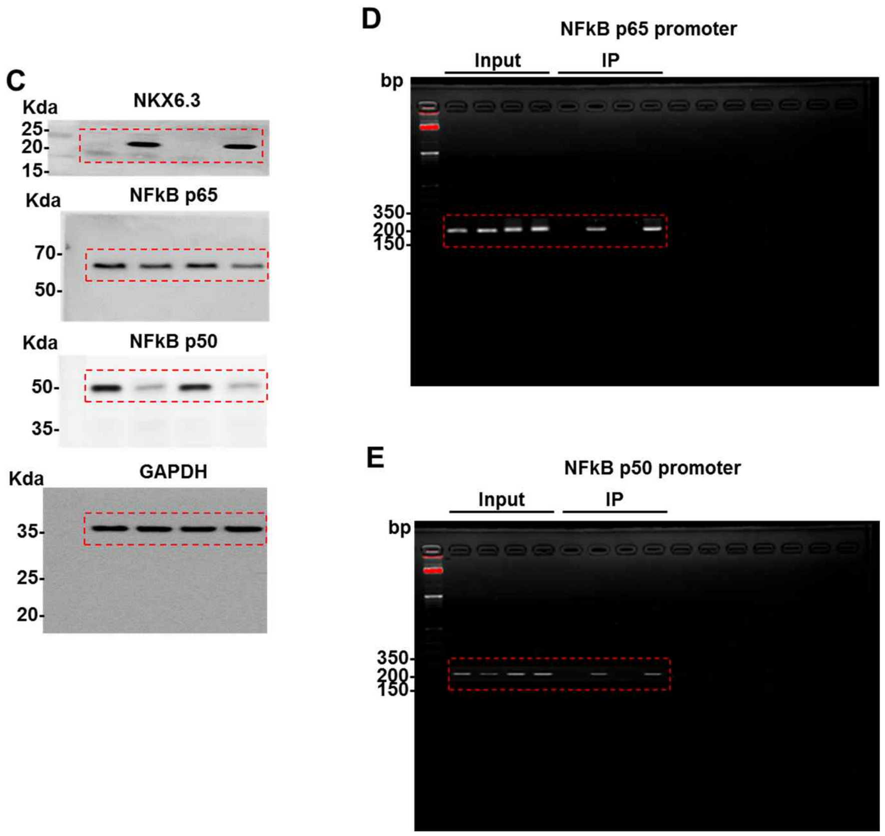

Supplementary Figure S6. Uncropped gel image for Figure 4.

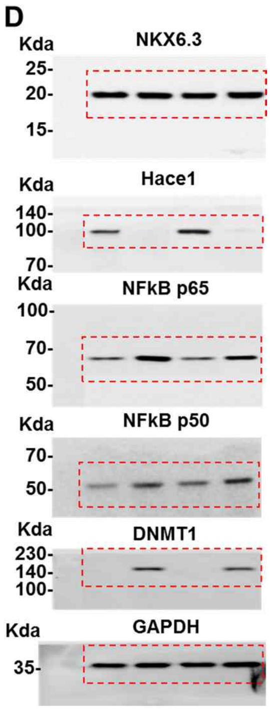

Supplementary Figure S7. Uncropped gel image for Figure 5.

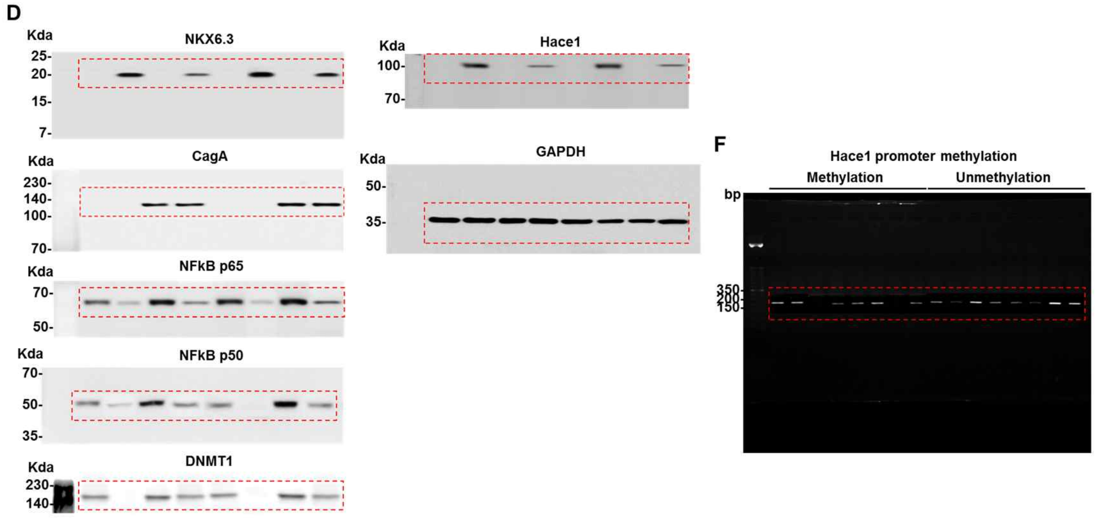

Supplementary Figure S8. Uncropped gel image for Figure 6.

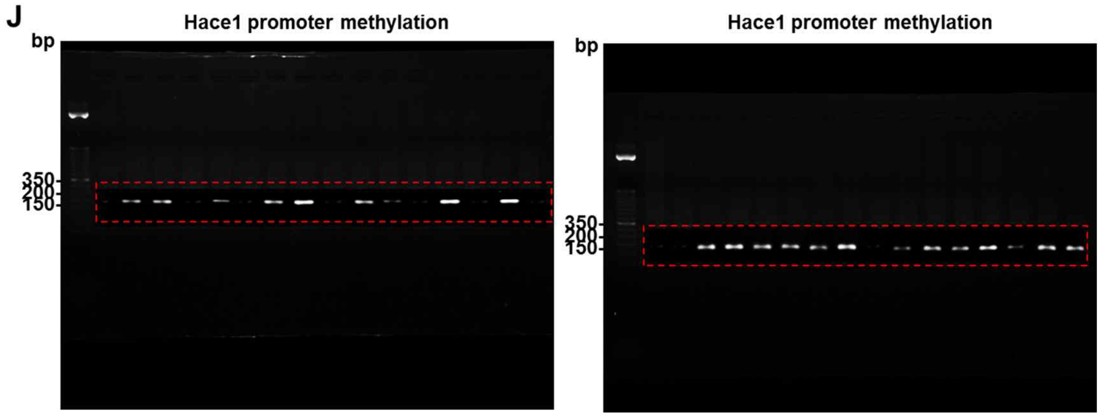

Supplementary Figure S9. Uncropped gel image for Figure 7.

Supplementary Table 1. List of primers used in this study

Gene name	primer sequence for real-time qPCR
NKX6.3	F: TCTTTCTGCTTCTGGGGTGT<br>R: GTCCAGCGGCTTGTTGTACT
Hace1	F: CCATTGTACTTTGCTTGCAGTCATGG<br>R: GAAGCAGCATGTTGATCTTGGCCTTT
DNMT1	F: CTACCAGGGAGAAGGACAGG<br>R: CTCACAGACGCCACATCG
NFκB p65	F: AGTACCTGCCAGATACAGACGAT<br>R: GATGGTGCTCAGGGATGACGTA
NFκB p50	F: AACAGAGAGGATTTCGTTTCCG<br>R: TTTGACCTGAGGGTAAGACTTCA
GSH	F: CTCTACGGCTCACCCAATGC<br>R: TCGTCGGATCACATGGATGTT
Nqo1	F: TGTATGACAAAGGACCCTTCC<br>R: CCCTTGCAGAGAGTACATGG
Ho-1	F: ACTTTCAGAAGGGCCAGGT<br>R: TGGGCTCTCCTTGTTGC
IL-6	F: AAGCCAGAGCTGTGCAGATGAGTA<br>R: TGTCCTGCAGCCACTGGTTC
IL-8	F: ATGACTTCCAAGCTGGCCGTGG<br>R: TTATGAATTCTCAGCCCTCTTC
TNFα	F: GGCAGTCAGATCATCTT CTCGAA<br>R: AAGAGGACCTGGGAGT AGATGA
β-actin	F: GTTGCTATCCAGGCTGTG<br>R: TGATCTTGATCTTCATTGTG

Promoter name	primer sequence for ChIP and ChIP-qPCR
DNMT1 promoter	F: ACCTCAGCCTCCCAAGTA<br>R: ATCGCTTGAGGTTAGGAGTT
NFκB p65 promoter	F: TCTGGGCCAGGTGTGGTGGC<br>R: TGGCCTCACAAAGTGCTGGGA
NFκB p50 promoter	F: ATTTTTTCCCAGTTACAGTGTTACTATT<br>R: ATTTTCCTAAGGTTTCATAATTAGTAAATA
Collagen	F: ATGGAGAAAGCAGCGAAGAA<br>R: AGCTGCTGTGGTGGAGAGAT

Primer name	primer sequence for Hace1 promoter MSP
Methylation	F: GAATGGAAGGTTTAATTTCGC<br>R: CTAAAACCCTACGTCAACCG
Unmethylation	F: TTGGAATGAATGGAAGGTTTAATTTT<br>R: CTAAAACCCTACATCAACCAACAA

Supplementary Table 2. List of antibodies used in this study

Name	Company	City
NKX6.3	Atlas antibodies	Stockholm, Sweden
p-Akt	Cell Signaling Technology	Beverly, MA, USA
Akt	Cell Signaling Technology	Beverly, MA, USA
Hace1	Santacruz	Santa Cruz, CA, USA
Nox1	Santacruz	Santa Cruz, CA, USA
Noxa1	Abcam	Cambrodge, MA, USA
p53	Cell Signaling Technology	Beverly, MA, USA
Nrf2	Cell Signaling Technology	Beverly, MA, USA
Catalase	Abcam	Cambrodge, MA, USA
mnSOD	Abcam	Cambrodge, MA, USA
DNMT1	Abcam	Cambrodge, MA, USA
NFκB p65	Santacruz	Santa Cruz, CA, USA
NFκB p50	Santacruz	Santa Cruz, CA, USA
CagA	Santacruz	Santa Cruz, CA, USA
GAPDH	Abcam	Cambrodge, MA, USA
